# Pharmacotherapy of psychiatric disorders in minority populations

**DOI:** 10.1192/j.eurpsy.2026.10398

**Published:** 2026-07-31

**Authors:** E. Döring-Brandl

**Affiliations:** Psychiatry, Psychotherapy and Psychosomatics, Alexianer Krankenhaus Hedwigshöhe, Berlin, Germany

## Abstract

**Abstract:**

Psychiatric treatment of patients with migration background can pose multiple challenges, including pharmacokinetic and pharmacodynamic factors influencing treatment response and occurrence of side effects, as well as differences in attitude towards psychiatric treatment options, cultural differences in symptom presentation, language barriers and stigmatization of mental disorders. The interplay of these factors can be complex and has to date only been partially understood. In this presentation, recent data regarding clinical, sociodemographic and biological factors that need to be considered in treatment of patients with migration background will be highlighted.

**Disclosure of Interest:**

E. Döring-Brandl Speakers bureau of: Speaker fees from Medice.

